# Connectivity-guided iTBS versus rTMS for treatment-resistant depression: Results from the BRIGhTMIND Study

**DOI:** 10.1192/j.eurpsy.2025.204

**Published:** 2025-08-26

**Authors:** M. Abdelghani

**Affiliations:** TMS Service, North London NHS Foundation Trust, London, United Kingdom

## Abstract

**Abstract:**

Treatment-resistant depression (TRD) remains a major clinical challenge, necessitating novel and more effective therapeutic approaches. The BRIGhTMIND study is the largest transcranial magnetic stimulation (TMS) clinical trial conducted in the UK. This multicentre, randomised controlled trial compares the efficacy of connectivity-guided intermittent theta burst stimulation (iTBS) with standard repetitive transcranial magnetic stimulation (rTMS) in patients with TRD. This talk will present key findings from the study, including response and remission rates, reported side effects, and key differences between the novel iTBS protocol tested and the conventional rTMS protocol used as the control condition. Additionally, we will explore the clinical implications of using functional connectivity to optimise stimulation targets. The results contribute to the growing evidence supporting TMS as an effective intervention for TRD and offer insights into the future of precision psychiatry in brain stimulation.

**Disclosure of Interest:**

None Declared

